# Supplementary material for: The mineralization characteristics of organic carbon and particle composition analysis in reconstructed soil with different proportions of soft rock and sand
Source: PeerJ. 2019 Sep 16;7:e7707. doi: 10.7717/peerj.7707 (PMC6752185; doi:10.7717/peerj.7707)
Supplement: Data S1 — CK: the volume ratio of soft rock to sand is 0:1; C1: the volume ratio of soft rock to sand is 1:5; C2: the volume ratio of soft rock to sand is 1:2; C3: the volume ratio of soft rock to sand is 1:1. [file peerj-07-7707-s001.docx]

**Regression equation raw data**

Mineralization rate (mg kg^-1^ d^-1^)

| Treatments | 1d | 2d | 3d | 4d | 5d | 8d | 11d | 14d | 18d | 22d | 26d | 30d |
| --- | --- | --- | --- | --- | --- | --- | --- | --- | --- | --- | --- | --- |
| CK | 28.11 | 34.96 | 49.61 | 16.94 | 37.75 | 6.66 | 5.65 | 4.84 | 3.51 | 3.63 | 3.35 | 3.41 |
| C1 | 58.67 | 42.04 | 49.61 | 33.64 | 24.20 | 13.39 | 11.94 | 14.12 | 12.59 | 13.13 | 11.74 | 10.18 |
| C2 | 43.51 | 33.00 | 25.65 | 23.23 | 30.01 | 14.04 | 18.08 | 15.97 | 8.96 | 10.16 | 7.31 | 7.97 |
| C3 | 48.16 | 55.98 | 66.07 | 39.93 | 56.14 | 38.24 | 24.44 | 27.27 | 19.66 | 20.69 | 13.12 | 13.48 |

Cumulative mineralization (mg kg^-1^)

| Treatments | 1d | 2d | 3d | 4d | 5d | 8d | 11d | 14d | 18d | 22d | 26d | 30d |
| --- | --- | --- | --- | --- | --- | --- | --- | --- | --- | --- | --- | --- |
| CK | 28.11 | 63.07 | 112.68 | 129.62 | 167.37 | 187.35 | 204.29 | 218.81 | 232.85 | 247.37 | 260.78 | 274.44 |
| C1 | 58.67 | 100.71 | 150.32 | 183.96 | 208.16 | 248.33 | 284.15 | 326.50 | 376.84 | 429.35 | 476.31 | 517.03 |
| C2 | 43.51 | 76.51 | 102.16 | 125.39 | 155.40 | 197.51 | 251.74 | 299.66 | 335.48 | 376.14 | 405.36 | 437.22 |
| C3 | 48.16 | 104.13 | 170.20 | 210.13 | 266.28 | 380.99 | 454.31 | 536.11 | 614.76 | 697.52 | 749.98 | 803.88 |
