# Supplementary material for: The mineralization characteristics of organic carbon and particle composition analysis in reconstructed soil with different proportions of soft rock and sand
Source: PeerJ. 2019 Sep 16;7:e7707. doi: 10.7717/peerj.7707 (PMC6752185; doi:10.7717/peerj.7707)
Supplement: Data S3 — Test plots 1 to 15 were set in 2009 and 16 to 30 were set in 2016. Plots 2, 7, 15, 17, 22 and 30 show the volume ratio of loess to sand is 1:2, plots 6, 10, 13, 21, 25 and 28 show the volume ratio of soft rock to sand is 1: 1, plots 5, 8, 9, 20, 23 and 24 show the volume ratio of soft rock to sand is 1: 2, plots 1, 4, 12, 16, 19 and 27 show the volume ratio of soft rock to sand is 1: 5, plots 3, 11, 14, 18, 26 and 29 show the volume ratio of soft rock to sand is 0:1. The color-marked test plots are selected for this trial. The red area represents CK treatment, the purple area represents C1 treatment, the blue area represents C2 treatment, and the yellow area represents C3 treatment. [file peerj-07-7707-s003.docx]

The red area represents CK treatment (the volume ratio of soft rock to sand is 0:1), the purple area represents C1 treatment (the volume ratio of soft rock to sand is 1:5), the blue area represents C2 treatment (the volume ratio of soft rock to sand is 1:2), and the yellow area represents C3 treatment (the volume ratio of soft rock to sand is 1:1).


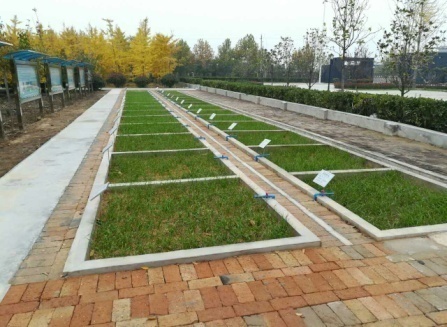

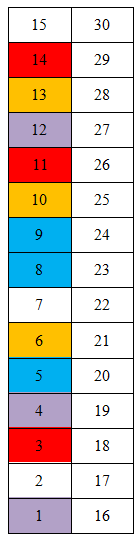

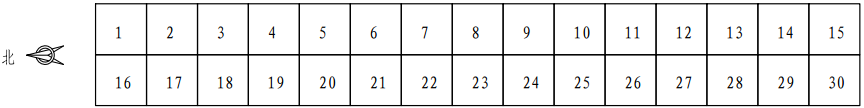


**18**

**17**

**16**

**3**

**1**

**2**

**North**

Each number corresponds to the test plot treatments

| Test plot number | 2, 7, 15 | 6, 10, 13 | 5, 8, 9 | 1, 4, 12 | 3, 11, 14 |
| --- | --- | --- | --- | --- | --- |
| Compound ratio | Loess: Sand =1:2 | Soft rock: sand =1:1 | Soft rock: sand =1:2 | Soft rock: sand =1:5 | Soft rock: sand =0:1 |
| Soil ages | 2009 | 2009 | 2009 | 2009 | 2009 |
| Test plot number | 17, 22, 30 | 21, 25, 28 | 20, 23, 24 | 16, 19, 27 | 18, 26, 29 |
| Compound ratio | Loess: Sand =1:2 | Soft rock: sand =1:1 | Soft rock: sand =1:2 | Soft rock: sand =1:5 | Soft rock: sand =0:1 |
| Soil ages | 2016 | 2016 | 2016 | 2016 | 2016 |
