# Supplementary material for: The mineralization characteristics of organic carbon and particle composition analysis in reconstructed soil with different proportions of soft rock and sand
Source: PeerJ. 2019 Sep 16;7:e7707. doi: 10.7717/peerj.7707 (PMC6752185; doi:10.7717/peerj.7707)

**The microstructure of compound soil in different proportions of soft rock and sand.** **Magnification is 1000 times.**

(a): the volume ratio of soft rock to sand is 0:1 (CK);


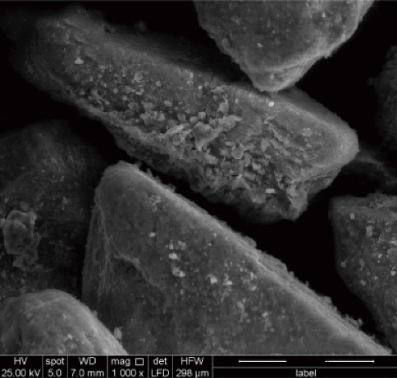


(b): the volume ratio of soft rock to sand is 1:5 (C1);


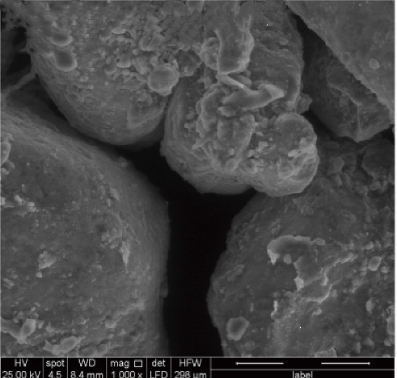


(c): the volume ratio of soft rock to sand is 1:2 (C2);


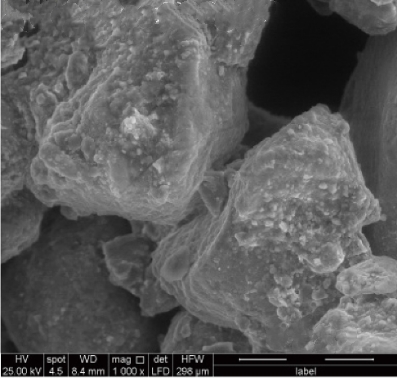


(d): the volume ratio of soft rock to sand is 1:1 (C3).


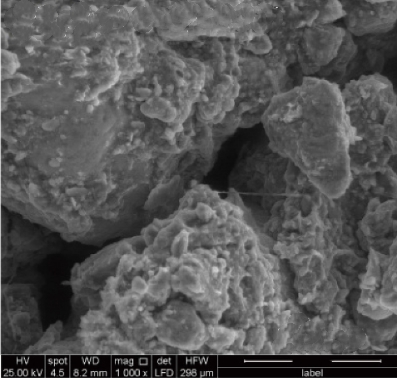

Supplement: Data S8 — (a): the volume ratio of soft rock to sand is 0:1 (CK); (b): the volume ratio of soft rock to sand is 1:5 (C1); (c): the volume ratio of soft rock to sand is 1:2 (C2); (d): the volume ratio of soft rock to sand is 1:1 (C3). The magnification is 1000 times. [file peerj-07-7707-s008.docx]
